# MALT1 Proteolytic Activity Suppresses Autoimmunity in a T Cell Intrinsic Manner

**DOI:** 10.3389/fimmu.2019.01898

**Published:** 2019-08-14

**Authors:** Annelies Demeyer, Ioannis Skordos, Yasmine Driege, Marja Kreike, Tino Hochepied, Mathijs Baens, Jens Staal, Rudi Beyaert

**Affiliations:** ^1^VIB Center for Inflammation Research, Ghent, Belgium; ^2^Department of Biomedical Molecular Biology, Ghent University, Ghent, Belgium; ^3^Center for Innovation and Stimulation of Drug Discovery (CISTIM), Leuven, Belgium

**Keywords:** autoimmunity, inflammation, Treg, Breg, CTLA-4, paracaspase, protease, lymphocyte

## Abstract

MALT1 is a central signaling component in innate and adaptive immunity by regulating NF-κB and other key signaling pathways in different cell types. Activities of MALT1 are mediated by its scaffold and protease functions. Because of its role in lymphocyte activation and proliferation, inhibition of MALT1 proteolytic activity is of high interest for therapeutic targeting in autoimmunity and certain lymphomas. However, recent studies showing that *Malt1* protease-dead knock-in (*Malt1*-PD) mice suffer from autoimmune disease have somewhat tempered the initial enthusiasm. Although it has been proposed that an imbalance between immune suppressive regulatory T cells (Tregs) and activated effector CD4^+^ T cells plays a key role in the autoimmune phenotype of *Malt1*-PD mice, the specific contribution of MALT1 proteolytic activity in T cells remains unclear. Using T cell-conditional *Malt1* protease-dead knock-in (*Malt1*-PDT) mice, we here demonstrate that MALT1 has a T cell-intrinsic role in regulating the homeostasis and function of thymic and peripheral T cells. T cell-specific ablation of MALT1 proteolytic activity phenocopies mice in which MALT1 proteolytic activity has been genetically inactivated in all cell types. The *Malt1*-PDT mice have a reduced number of Tregs in the thymus and periphery, although the effect in the periphery is less pronounced compared to full-body *Malt1*-PD mice, indicating that also other cell types may promote Treg induction in a MALT1 protease-dependent manner. Despite the difference in peripheral Treg number, both T cell-specific and full-body *Malt1*-PD mice develop ataxia and multi-organ inflammation to a similar extent. Furthermore, reconstitution of the full-body *Malt1*-PD mice with T cell-specific expression of wild-type human *MALT1* eliminated all signs of autoimmunity. Together, these findings establish an important T cell-intrinsic role of MALT1 proteolytic activity in the suppression of autoimmune responses.

## Introduction

MALT1 (PCASP1 or paracaspase-1) is ubiquitously expressed in multiple cell types, where it is involved in signaling leading to pro-inflammatory gene expression. The best known role of MALT1 is the activation of NF-κB signaling in response to T cell and B cell antigen receptor triggering, which is critical for the adaptive immune system. At the border between the innate and adaptive immune system, MALT1 acts downstream of several innate immune receptors on antigen-presenting dendritic cells (DC), which in turn regulate cytokine production that can influence the polarization of the adaptive immune system. MALT1 is also critical for NF-κB induction, but not for degranulation responses, downstream of several ITAM-coupled natural killer cell (NK) receptors and the Fcε receptor on mast cells (1). MALT1-deficient mice and patients show primary immunodeficiency (PID) via defects in T and B cells, in particular marginal zone (MZ) B cell development and antigen-induced T cell proliferation and activation, which leads to immunodeficiency and failure to respond to vaccination (2–8). A MALT1-deficient patient suffering from recurrent infections was cured by transplantation of mostly T cells (8). This indicates a major role for T cells as a determinant of the compromised immunity phenotypes caused by MALT1 deficiency. T cells are highly dependent on antigen receptor signaling, which requires MALT1. In contrast, B cells can be alternatively activated by innate receptors in a MALT1-independent manner. This might explain the lower phenotypic impact of MALT1 deficiency in B cells (2, 6, 7, 9). MALT1 signaling in non-lymphoid cells is also critical for optimal immunity, as illustrated by PID patients susceptible to fungal infections due to a CARD9 mutant that fails to recruit downstream BCL10 and MALT1 (10). Apart from its role in immune signaling through loss-of-function, aberrant activation or expression of MALT1 has been associated to different forms of B cell cancer, such as ABC-DLBCL (11–14), low-grade MALT lymphomas (15–19), and a complex type of PID called B cell expansion with NF-κB and T cell anergy (BENTA) (20, 21).

MALT1 belongs to the type 1 paracaspase family, an ancient protein family that originated before the last common ancestor of planulozoa (bilaterians and cnidarians) (22, 23). The type 1 paracaspases consist of an amino-terminal death domain, immunoglobulin domains and a caspase-like domain. Upon activation, MALT1 acts as a scaffold and recruits critical downstream signaling components such as TRAF6 and TAK1 in order to activate NF-κB and JNK/AP-1 dependent transcription (2, 3, 24, 25). The caspase-like domain has received plenty of research attention after it was established that paracaspases have protease activity (26, 27). Both the scaffold function leading to NF-κB and the protease activity of MALT1 are of critical importance, since they have been functionally conserved at least as far back as the last common ancestor of the three type 1 paracaspase paralogs in jawed vertebrates (22, 23). An ever growing list of paracaspase protease substrates—which at this moment includes auto-processing (28–30), adaptor proteins (27), transcription factors (31), deubiquitinases (26, 32), ubiquitin ligases (33–35) and specific RNA degrading enzymes (36–38)—illustrate that MALT1 protease activity removes inhibitory proteins and fine-tunes the type of responses that are induced (1, 39). There is only a single member of the paracaspase family in mammals (22). This, as well as the signal fine-tuning by an enzymatic activity, makes MALT1 protease activity a very attractive therapeutic target in humans for the treatment of autoimmunity and some cancers. At this moment, several independent studies have described active site or allosteric MALT1 protease inhibitors (11–14, 40–45), and mouse studies have already indicated the therapeutic value of some of these inhibitors in certain types of cancer (13, 14, 43, 46) and inflammatory diseases (40). Recently, four independent papers reported unexpected autoimmune phenotypes such as multi-organ inflammation, including the stomach, and ataxia in full-body *Malt1*-PD mice (4–6, 47), which could indicate a risk when inhibiting MALT1 protease activity for therapeutic reasons. While at first sight counter-intuitive, autoimmune or autoinflammatory diseases (AID) are in fact common in patients suffering from PID (48). The general conclusion from the different *Malt1*-PD mouse studies was that the disease was caused by an imbalance between effector CD4^+^ T cells and Tregs (4, 5). Much of the disease could be mitigated by deletion of IFN-γ in the *Malt1*-PD mice, indicating that IFN-γ is a primary driver of disease (5). However, another report also suggested a contribution from immune-regulatory IL-10 producing B-cells (Bregs) (47). Moreover, MALT1 also regulates the activation of DC cells, NK cells and mast cells (1), indicating the possibility that MALT1 activity in some of these cells may also regulate inflammatory T and B cell responses associated with autoimmunity in *Malt1*-PD mice. Given the complexity of MALT1 function in the development of lymphoid cells and activation of several immune cells, the study of cell-intrinsic functions of MALT1 requires *Malt1* conditional knock-in (KI) mice. In order to better predict safety risks upon pharmacological MALT1 protease inhibition and get further insight into the role of T cells in the autoimmune pathology of *Malt1*-PD mice, we employed conditional KI mice lacking MALT1 proteolytic activity specifically in T cells (*Malt1*-PDT), and compared their phenotype with mice lacking MALT1 proteolytic activity in all cell types (*Malt1*-PD). To verify that only MALT1 protease deficiency in T cells can cause the disease, we also reconstituted *Malt1*-PD mice with transgenic T cell-specific expression of human wild-type (WT) *MALT1*. Taken together, we show that T cell-specific inactivation of MALT1 proteolytic activity phenocopies inactivation of MALT1 in all cell types, resulting in a disruption in T cell immune homeostasis and development of autoimmunity, indicating an important T cell-intrinsic role of MALT1 proteolytic activity in keeping normal immune homeostasis.

## Results

### T Cell-Specific Loss of MALT1 Proteolytic Activity Is Sufficient to Cause Ataxia and Weight Retardation to the Same Extent as Full-Body Loss of MALT1 Proteolytic Activity

**Figure 1:**
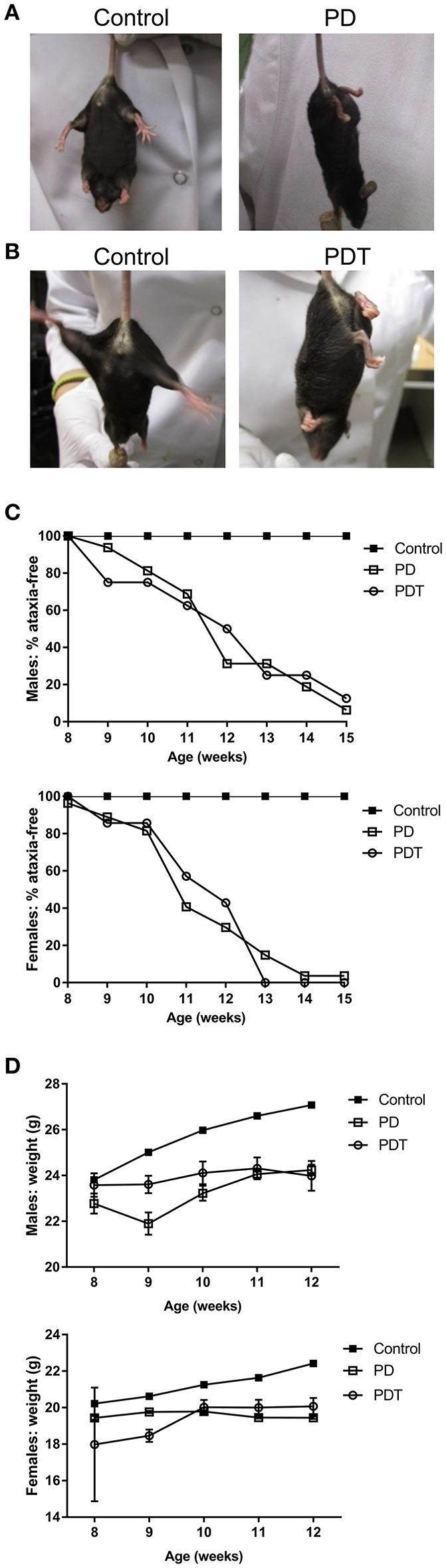
*Malt1-*PD and *Malt1*-PDT mice display ataxia and weight retardation. **(A,B)** Representative pictures of *Malt1*-PD and -PDT mice suffering from ataxia, characterized by leg clutching. **(C)** Ataxia onset in male and female control, *Malt1*-PD and -PDT mice monitored from week 8 to 15 (male mice: control *n* = 41, *Malt1*-PD *n* = 16, and *Malt1*-PDT *n* = 8; female mice: control *n* = 37, *Malt1*-PD *n* = 26 and *Malt1*-PDT *n* = 7). **(D)** Weight retardation in male and female *Malt1*-PD and -PDT mice monitored from week 8-12 until termination. Values after week 12 are excluded due to a too low number of surviving *Malt1*-PDT mice, causing very broad 95% confidence intervals. Male mice: week 8: control *n* = 41, *Malt1*-PD *n* = 9 and *Malt1*-PDT *n* = 6; week 9: control *n* = 49, *Malt1*-PD *n* = 8 and *Malt1*-PDT *n* = 8; week 10: control *n* = 50, *Malt1*-PD *n* = 11 and *Malt1*-PDT *n* = 7; week 11: control *n* = 61, *Malt1*-PD *n* = 16 and *Malt1*-PDT *n* = 8; week 12: control *n* = 59, *Malt1*-PD *n* = 16 and *Malt1*-PDT *n* = 7 and female mice: week 8: control *n* = 37, *Malt1*-PD *n* = 10 and *Malt1*-PDT *n* = 3; week 9: control *n* = 49, *Malt1*-PD *n* = 18 and *Malt1*-PDT *n* = 6; week 10: control *n* = 50, *Malt1*-PD *n* = 23 and *Malt1*-PDT *n* = 5; week 11: control *n* = 56, *Malt1*-PD *n* = 27 and *Malt1*-PDT *n* = 6; week 12: control *n* = 54, *Malt1*-PD *n* = 26 and *Malt1*-PDT *n* = 6.

To characterize the contribution of hematopoietic cells in the autoimmune phenotype of *Malt1*-PD mice, all previous studies have relied on crosses with other knock-out (KO) lines (e.g., IFN-γ KO), bone marrow transplants and injection of WT Tregs (4–6, 47). However, none of these experiments allow an undeniable conclusion on the specific role of T cells in the different autoimmune responses observed in *Malt1*-PD mice. In order to further characterize this phenotype, we generated T cell-specific *Malt1*-PDT mice and compared their phenotype with that of full-body *Malt1*-PD mice that we also generated. We used the F1 offspring from the *Malt1*^*FL*/*FL*^ *CD4-Cre X Malt1*^*PD*/+^ cross, where the *Malt1*^*PD*/*FL*^ *CD4-Cre* offspring represent *Malt1*-PDT mice. The remaining F1 offspring (*Malt1*^PD/FL^, *Malt1*^+/FL^, and *Malt*^+/*FL*^ *CD4-Cre*) all behaved as WT and were consequently all used as phenotypic controls (Supplemental Figures 1A–C). To obtain a positive phenotypic control, *Malt1*^*PD*/+^ were crossed with *Malt1*^−*LacZ*/−*LacZ*^ KO mice, where *Malt1*^PD/−LacZ^ F1 offspring represent full-body *Malt1-*PD mice and *Malt1*^+/−*LacZ*^ offspring represent healthy controls with the same gene dosage as our *Malt1-*PDT mice (Supplemental Figures 1A–C). Just like in the full-body *Malt1*-PD mice, the T cell-specific *Malt1*-PDT mice displayed a weight retardation, starting from approximately 9 weeks of age, and ataxia, starting on average at 11–12 weeks of age (Figures 1A–D). This is also similar to what was previously observed for other full-body *Malt1*-PD mice in several independent studies (4–6, 47). A WT mouse will stretch its hind legs when suspended by its tail to maintain its balance (Figure 1A). A mouse suffering from ataxia will flail around with its hind legs at an early stage of disease and start clutching its hind paws (and sometimes also front paws) at a more advanced stage (Figures 1A,B). We scored a mouse as having ataxia at the moment the hind paws were completely clutched while hanging by the tail. Although time of onset was not different (Figures 1C,D), we noticed that the overall symptoms were more severe in females than in males, which is similar to what has been previously observed in an independent *Malt1*-PD mouse line (47). This observation is also consistent with the sexual dimorphism in autoimmune diseases seen in humans (49, 50). Since the T cell-specific *Malt1*-PDT mice show as severe disease development as the full-body *Malt1*-PD mice, we can conclude that absence of MALT1 protease activity in T cells only is sufficient to induce ataxia and weight loss to a similar extent as induced by its absence in all body cells.

### T Cell-Specific Loss of MALT1 Proteolytic Activity Causes Multi-Organ Inflammation

**Figure 2:**
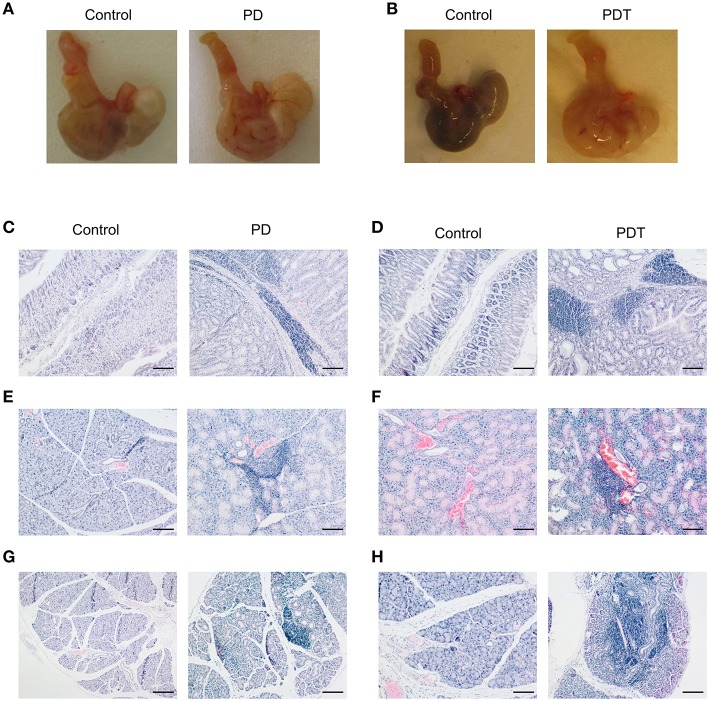
Multi-organ inflammation in *Malt1*-PD and *Malt1*-PDT mice. **(A,B)** Representative pictures of stomach of *Malt1-*PD and -PDT mice with increased vascularization compared to their corresponding controls. **(C,D)** H&E staining showing immune infiltration in the submucosa of the stomach of *Malt1-*PD **(C)** and *Malt1*-PDT **(D)** mice. Scale bar 100 μm. **(E,F)** H&E staining showing immune infiltration in the salivary gland of *Malt1*-PD **(E)** and *Malt1*-PDT **(F)** mice. Scale bar 100 μm. **(G,H)** H&E staining showing immune infiltration in the lacrimal gland of *Malt1*-PD **(G)** and *Malt1*-PDT **(H)** mice. Scale bar 100 μm.

**Figure 3:**
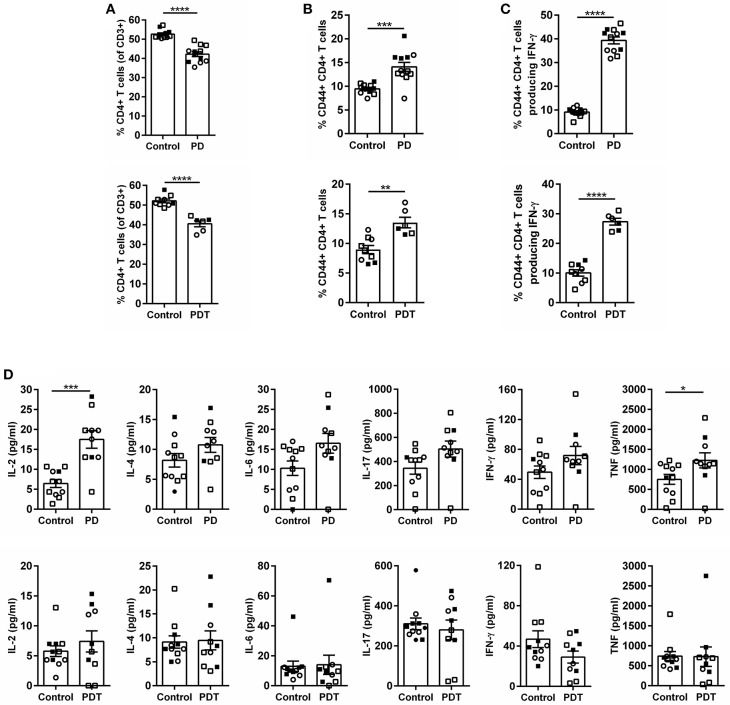
Inflammation in *Malt1*-PD and *Malt1*-PDT mice is local and not systemic. **(A)** CD4^+^ T cells in *Malt1*-PD (top panel) and *Malt1*-PDT (bottom panel) mice. **(B)** CD44^+^CD4^+^ T cells in *Malt1*-PD (top panel) and *Malt1*-PDT (bottom panel) mice. **(C)** CD44^+^CD4^+^ T cells producing IFN-γ in *Malt1*-PD (top panel) and *Malt1*-PDT (bottom panel) mice. For **(A–C)** lymphocytes from cLN were stimulated for 4 h with PMA/ionomycin and brefeldin A to determine the different cell populations by flow cytometry. *Malt1*-PD mice: *n* = 11, corresponding control mice: *n* = 12; *Malt1*-PDT mice: *n* = 6, corresponding control mice: *n* = 9. **(D)** Serum levels of IL-2, IL-4, IL-6, IL-17, IFN-γ, and TNF in *Malt1*-PD (top panels) and *Malt1*-PDT (bottom panels) mice. Data represent three separate serum collections: collection 1 = filled squares, collection 2 = open squares and collection 3 = open circles. *Malt1*-PD mice: *n* = 10, corresponding control mice: *n* = 11 and *Malt1*-PDT mice: *n* = 11, corresponding control mice: *n* = 10. The mean ± SEM is indicated on the graphs. The statistical significance between groups was calculated with an unpaired 2 tailed Student's *t-*test: ^*^p < 0.05, ^**^p < 0.01, ^***^p < 0.001, and ^****^p < 0.0001.

After birth, mice were checked regularly and no external signs of suffering could be observed before the development of ataxia. However, upon sacrifice we noticed that the stomach of *Malt1*-PD and -PDT mice was more rigid and vascularised (Figures 2A,B). By means of H&E staining we also observed severe gastritis, plus immune infiltration in lacrimal and salivary glands indicating that the mice already had developed inflammation in other organs (Figures 2C–H). In order to minimize the suffering of mice, we euthanized them as soon as possible after the observation of ataxia. To assess whether disease development in *Malt1*-PD and -PDT mice was due to the same mechanism, we evaluated cytokine production by CD44^+^ CD4^+^ T cells in cervical lymph nodes (cLN) of mice already suffering from ataxia. We decided to analyze cLN as they are located close to known inflamed organs. Both *Malt1*-PD and -PDT mice have a slight reduction in total CD4^+^ T cells and an increase in CD4^+^ T cells that express CD44, so-called effector CD4^+^ T cells compared to WT control mice (Figures 3A,B). We also observed a strong upregulation of IFN-γ-producing CD44^+^CD4^+^ T cells in *Malt1*-PD and -PDT mice, indicating a T cell intrinsic role for MALT1 protease activity in restricting Th1 responses (Figure 3C). We further assessed serum cytokine levels in *Malt1*-PD and -PDT mice. For IL-2 and TNF there was a significant, but mild increase in the *Malt1*-PD compared to control mice, which was no longer detectable in *Malt1*-PDT mice. No increase in IL-4, IL-6, IL-17 and IFN-γ could be detected in serum of *Malt1*-PD and -PDT mice (Figure 3D). Therefore, we can conclude that both *Malt1*-PD and -PDT mice do not suffer from systemic inflammation, but instead suffer from local Th1-associated multi-organ inflammation.

### A T Cell-Intrinsic Role for MALT1 Proteolytic Activity Is Critical for Thymic nTreg Development

**Figure 4:**
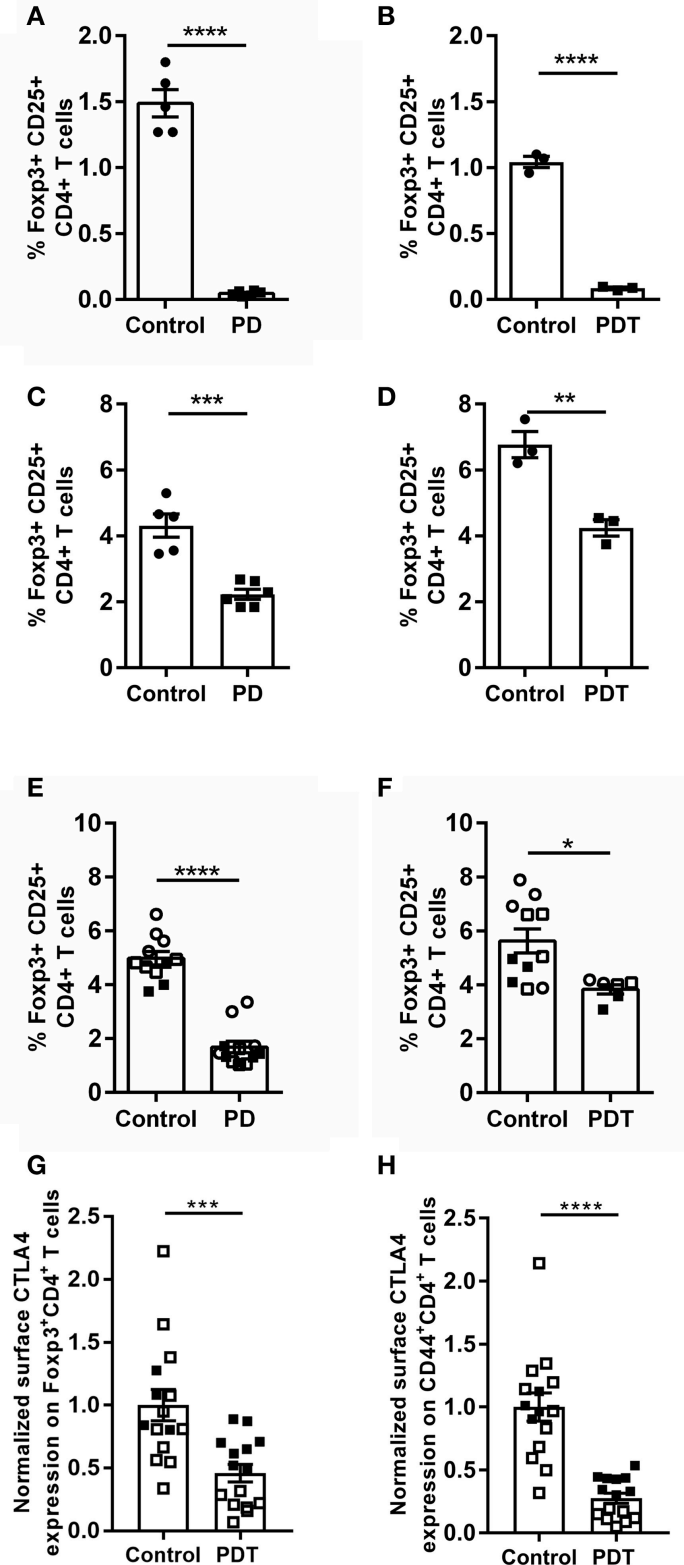
Reduced Treg frequency and reduced surface CTLA-4 expression on Tregs and effector CD4^+^ T cells in *Malt1*-PD and *Malt1*-PDT mice. **(A,B)** Treg frequency (Foxp3^+^CD25^+^CD4^+^CD8^−^ T cells) in thymus of young *Malt1*-PD (*n* = 6) **(A)** and *Malt*-PDT (*n* = 3) **(B)** mice and their corresponding controls (*n* = 5 and *n* = 3, respectively). **(C,D)** Treg frequency in cLN of young *Malt1-*PD (*n* = 6) **(C)** and *Malt1*-PDT (*n* = 3) **(D)** mice and their corresponding controls (*n* = 5 and *n* = 3, respectively). **(E,F)** Treg frequency in *Malt1*-PD (*n* = 11) **(E)** and *Malt1*-PDT (*n* = 6) **(F)** mice suffering from ataxia and their corresponding controls (*n* = 12 and *n* = 9, respectively). Lymphocytes were stimulated for 4 h with PMA/ionomycin and the data represent three individual experiments: experiment 1 = filled squares, experiment 2 = open squares and experiment 3 = open circles. **(G,H)** Normalized CTLA-4 expression on the surface of Tregs **(G)** and CD44^+^CD4^+^ T cells **(H)** from young disease free *Malt1*-PDT mice (*n* = 15) and their corresponding controls (*n* = 15). The individual percentages of Foxp3^+^CD4^+^ T cells or CD44^+^CD4^+^ T cells that express CTLA-4 on their surface is normalized against the average percentage of the corresponding control mice of each individual experiment. Lymphocytes were stimulated for 4 h with PMA/ionomycin and data represent two individual experiments: experiment 1 = filled squares, experiment 2 = open squares. For **(A–H)**: all data were obtained via flow cytometry. The mean ± SEM is indicated on the graphs. The statistical significance between groups was calculated with an unpaired 2 tailed Student's *t-*test: ^*^p < 0.05, ^**^p < 0.01, ^***^p < 0.001, and ^****^p < 0.0001.

The best known Tregs are Foxp3^+^CD25^+^CD4^+^ T cells (51), which have two distinct developmental origins. Some develop in the thymus at a young age—the so-called natural Tregs (nTregs). Others mature in the periphery from naïve conventional T cells during extended exposure to antigen or under inflammatory conditions—the so-called induced Tregs (iTregs). Both populations are genetically distinct and have non-redundant functions (52, 53). MALT1 has been shown to be specifically required for thymic Treg development, while induced peripheral Treg formation in aged mice is not inhibited by MALT1 deficiency (4, 5, 54). The ability to induce Treg formation in *Malt1*-KO mice is also consistent with previous *in vitro* differentiation studies using a high dose of anti-CD3 to stimulate the TCR (55). This might indicate a threshold effect which is influenced by MALT1. Therefore, we investigated the role of MALT1 proteolytic activity in thymic Treg development in young healthy (ataxia-free) *Malt1*-PD and -PDT mice. A clear defect in thymic Treg development could be observed, since Foxp3^+^CD25^+^CD4^+^ T cells were almost absent in the thymus of young *Malt1*-PD and -PDT mice (Figures 4A,B). Furthermore, thymic Treg development could be rescued upon transgenic expression of human WT MALT1 in cells targeted by *CD4-Cre* (Figures 4D,E). This clearly indicates a T cell-intrinsic role for MALT1 protease activity in nTreg development.

Tregs are present in the cLN of young *Malt1*-PD and -PDT mice, albeit at lower levels than in control mice (Figures 4C,D). The higher Treg numbers found in cLN of young *Malt1*-PDT mice could be a secondary effect from inflammation-induced formation of iTregs or peripheral proliferation of the few remaining nTregs. The nearly absent levels of Tregs in thymus, but detectable levels of Tregs in the periphery is a common feature of *Malt1-*PD and *-*PDT mice, as well as *Malt1*-KO mice (4, 5, 47, 54, 56).

**Figure 5:**
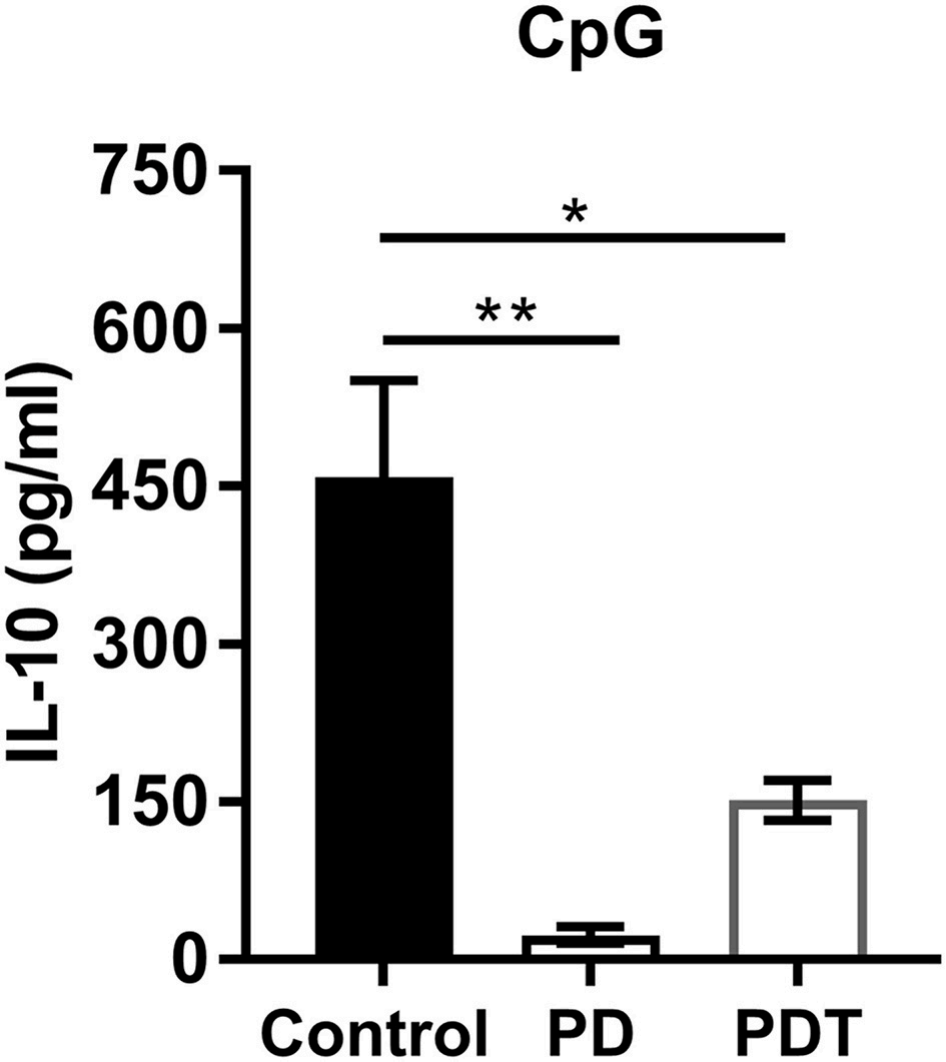
Bregs are almost absent in *Malt1*-PD mice, but are present in *Malt1*-PDT mice. IL-10 secretion by control (*n* = 4), *Malt1*-PD (*n* = 3), and *Malt1*-PDT (*n* = 3) splenic B cells after overnight stimulation with TLR9 ligand CpG. The mean ± SEM is indicated on the graphs. The statistical significance between groups was calculated with an unpaired 2 tailed Student's *t-*test: ^*^p < 0.05 and ^**^p < 0.01.

In addition to a decrease in Tregs, also a decrease in the amount of Bregs was suggested to contribute to the autoimmune disease phenotype in *Malt1*-PD mice (47). Bregs can originate from many different B cell lineages, where marginal zone (MZ) B cells serve as an important source in the spleen (57). MZ B cells are disrupted in *Malt1*-KO and *Malt1-*PD mice. We therefore investigated MZ B cell development in T cell-specific *Malt1*-KO and *Malt1*-PDT mice compared to WT and full-body *Malt1*-PD and -KO mice. In contrast to full body *Malt1*-PD and -KO mice, MZ B cell development is completely normal in both T cell-specific *Malt1*-KO and *Malt1*-PDT mice (Supplemental Figures 2A–D). Furthermore, in contrast to *Malt1*-PD mice, Bregs were not absent in the *Malt1*-PDT mice (Figure 5). Together, these findings argue against a contribution from a decrease in IL-10 producing Bregs to the autoimmune phenotype.

### Loss of MALT1 Proteolytic Activity Reduces Expression of the Treg Functionality Marker CTLA-4

Previously Jaworski et al. (4) and Bornancin et al. (47) reported a reduction of peripheral Tregs in *Malt1*-PD mice. We could confirm this in cLN from young disease-free *Malt1-*PD mice and in mice already suffering from ataxia. Notably, at the time of sacrifice, when the mice had developed ataxia, the number of peripheral Tregs in *Malt1*-PDT mice is higher than in *Malt1*-PD mice (Figures 4E,F), indicating that also MALT1 protease-dependent signals from non-T cells may promote peripheral Treg induction. Nevertheless, both *Malt1*-PD and -PDT mice developed equally severe disease symptoms, indicating that disease development in the *Malt1*-PD and -PDT cannot be solely explained by reduced peripheral iTreg levels. Therefore, we assessed the functionality of Tregs by means of their surface expression of CTLA-4, which binds CD80 and CD86 proteins found on the surface of antigen presenting cells. These proteins provide, by binding to CD28 on the T cells, the costimulatory signal needed for optimal T cell activation. By expressing CTLA-4, Tregs can downregulate CD80 and CD86 expression on antigen presenting cells, leading to reduced T cell proliferation, survival and IL-2 production (58). We have previously observed, via flow cytometry on splenocytes, that the percentage of Foxp3^+^CD4^+^ T cells that express CTLA-4 on their surface is reduced in *Malt1*-KO mice compared to WT mice (unpublished data). To assess the effect of MALT1 proteolytic activity on the percentage of Tregs (Foxp3^+^CD4^+^ T cells) with surface CTLA-4 expression, we isolated cells from cLN of young disease-free *Malt1*-PDT mice and analyzed CTLA-4 surface expression by flow cytometry. This revealed that the percentage of surface CTLA-4 expressing Tregs was also reduced in *Malt1*-PDT mice compared to control mice (Figure 4G). The reduced expression of CTLA-4 is not the result of a generalized defect in effector Treg differentiation, since expression of other Treg effector markers is not affected (Supplemental Figures 3A–D). CTLA-4 can also be expressed on effector CD4^+^ T cells (CD44^+^CD4^+^ T cells), where reduced expression is associated with elevated tissue infiltration and suppression of autoreactivity (59, 60). As for the Tregs, also here the percentage of CTLA-4 surface-positive CD44^+^CD4^+^ T cells was found to be reduced in young disease-free *Malt1*-PDT mice (Figure 4H). Together, these results clearly demonstrate that MALT1 proteolytic activity in T cells regulates CTLA-4 surface expression in Tregs and CD44^+^CD4^+^ T cells, indicating that MALT1 proteolytic activity influences Treg functionality in a T cell-intrinsic manner, since even a moderate decrease in CTLA-4 expression can lead to autoimmunity in humans (61). The reduced CTLA-4 expression could thus be an important factor regulating autoimmunity in the *Malt1*-PDT mice.

### T Cell-Specific Transgenic Human MALT1 Reconstitution Rescues MALT1 Protease-Deficient Mice From Autoimmune Disease

**Figure 6:**
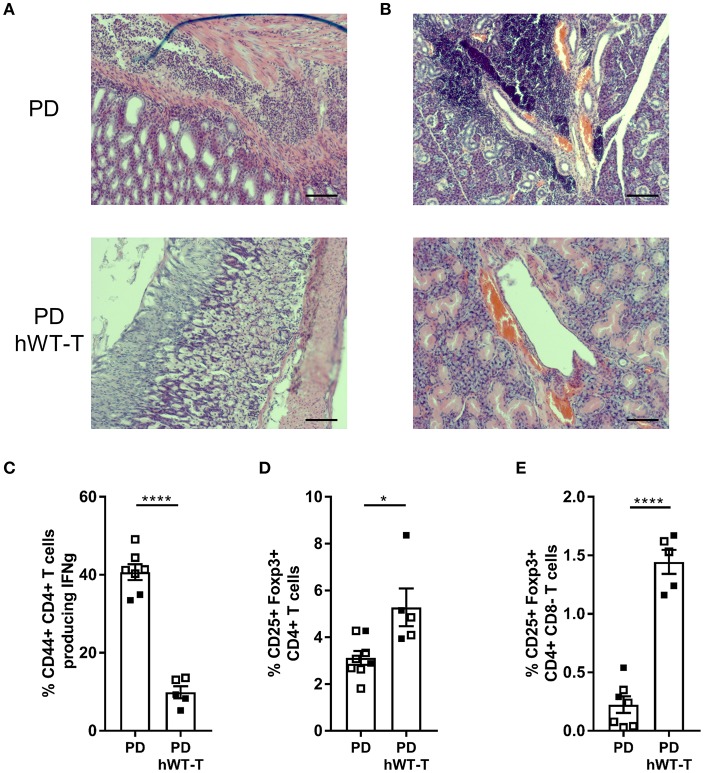
Expression of hMALT1 in T cells of *Malt1*-PD mice rescues the disease phenotype. **(A,B)** H&E staining of stomach **(A)** and salivary gland **(B)** of *Malt1*-PD (PD) and *Malt1*-PD mice expressing wild type human MALT1 in T cells (*Malt1*^*PD*/*-*^ *Rosa26*^*LSL-MALT1-WT*^ *CD4-Cre* = PD hWT-T) mice. **(C)** Percentage of CD44^+^CD4^+^ T cells producing IFN-γ in PD and PD hWT-T mice. **(D)** Treg frequency (Foxp3^+^CD25^+^CD4^+^CD8^−^ T cells) in thymus of PD and PD hWT-T mice. **(E)** Treg frequency in cLN of PD and PD hWT-T mice. For **(C–E)**: all data were obtained via flow cytometry and for **(E)**, lymphocytes were stimulated for 4 h with PMA/ionomycin. Data represent two individual experiments: experiment 1 = open squares and experiment 2 = filled squares. *Malt1*-PD mice: total *n* = 7 and *Malt1*-PD hWT-T mice: total *n* = 5, age 13–18 weeks. The mean ± SEM is indicated on the graphs. The statistical significance between groups was calculated with an unpaired 2 tailed Student's *t*-test: ^*^p < 0.05 and ^****^p < 0.0001.

Since both full-body *Malt1*^PD/−^ and T cell-specific *Malt1*^PD/FL^ *CD4-Cre* mice equally develop an inflammatory phenotype, we can conclude that the loss of MALT1 proteolytic activity in cells targeted by *CD4-Cre* is sufficient to induce a full autoimmune response. To verify that MALT1 enzymatic activity in other cells not targeted by *CD4-Cre* may still contribute to the development of autoimmune disease, we generated *Malt1*^*PD*/−^ *Rosa26*^*LSL-MALT1-WT*^ *CD4-Cre* mice in which all cells endogenously express protease-dead MALT1, and where human WT MALT1 is specifically expressed as a transgene in cells that have experienced *CD4-Cre* activity. These mice did no longer show gastritis and immune infiltration in lacrimal and salivary glands as observed in *Malt*-PD mice (Figures 6A,B), nor did they show an increase in IFN-γ producing CD4^+^ T cells (Figure 6C). Moreover, transgenic expression of WT MALT1 in cells targeted by *CD4-Cre* in a *Malt1*-PD background suppressed the strong drop in the amount of Tregs in the thymus and cLN of *Malt1*-PD mice (Figures 6D,E). The complete rescue of the autoimmune phenotype in full-body *Malt1*-PD mice by T cell-specific WT MALT1 expression further demonstrates that only T cell MALT1 protease activity is critical to suppress the autoimmune phenotype.

## Discussion

MALT1 proteolytic activity plays a key role in both immune cells and non-immune cells (62, 63), and mice expressing a catalytically inactive MALT1 mutant (*Malt1*-PD mice) were previously shown to suffer from severe autoimmunity (4–6, 47). Conditional genetic models are essential to determine the critical cell type responsible for disease development in *Malt1*-PD mice. We studied the T cell-intrinsic functions of MALT1 proteolytic activity in the regulation of immune and autoimmune responses by comparing *Malt1*-PD mice with *Malt1*-PDT mice that specifically express catalytically inactive MALT1 in T cells. We report that both lines develop similar disease symptoms and autoimmunity, indicating that MALT1 proteolytic activity suppresses autoimmunity in a T cell-intrinsic manner. Autoimmune disease in *Malt1*-PD mice was previously explained by a disrupted balance between effector CD4^+^ T cells and the number of Tregs (4, 5, 47). Both *Malt1*-PD and *Malt1*-PDT mice have almost no nTregs in their thymus, indicating a T cell-intrinsic role of MALT1 protease activity in thymic Treg development. nTregs inhibit autoreactive T cells to become mature Th1 cells (53, 64). If induced peripheral Tregs cannot compensate for the lack of thymus-derived nTregs, this may explain the Th1-dependent autoimmune phenotype of *Malt1*-PD and *Malt1*-PDT mice. Therefore, it might be interesting to investigate whether inducible genetic inactivation of MALT1 proteolytic activity in adult animals results in a similar phenotype. Moreover, less well-known Foxp3-negative regulatory T cell populations (e.g., Tr1 and Th3 cells) (52) may also play a role. Of note, the reduction of Foxp3^+^ Tregs is less pronounced in *Malt1*-PDT mice compared to *Malt1*-PD mice, suggesting that also MALT1 protease-dependent signals from cells not targeted by *CD4-Cre* contribute to the decrease in Tregs. One non-T cell population that could influence Treg numbers and which is different between *Malt1*-PD and *Malt1-*PDT mice are the Breg cells (65, 66).

As *Malt1*-PD and *Malt1*-PDT mice develop equally severe disease symptoms, disease development cannot be solely explained by reduced peripheral Treg levels. Therefore, we also assessed the Treg surface expression of CTLA-4, which is a known Treg functionality marker and whose deficiency partially leads to similar disease symptoms as those seen in *Malt1*-PD and -PDT mice (64, 67–70). In addition, *in vivo* models for colitis and diabetes demonstrate the importance of CTLA-4 for the Treg inhibitory function (69). It is believed that the inhibitory effect of Tregs occurs via CTLA-4 mediated trans-endocytosis of CD80 and CD86 proteins from antigen presenting cells (71), which impairs CD28 co-stimulation that is required for optimal T cell activation (58, 71). We found that the number of Tregs that expressed CTLA-4 on their surface was much lower in *Malt1*-PDT mice compared to WT controls. We therefore propose that not the reduced number of Treg *per se*, but rather impaired Treg functionality is responsible for autoimmune disease development in *Malt1*-PDT mice. Of note, also the number of effector CD4^+^ T cells expressing CTLA-4 on their surface was found to be reduced in *Malt1*-PDT mice compared to controls. CTLA-4 on effector CD4^+^ T cells can also inhibit T cell responses in a cell extrinsic way, but their suppressive capacities are modest compared to Tregs (72, 73). Nevertheless, reduced CTLA-4 expression on effector CD4^+^ T cells might also contribute to the disease phenotype observed in our *Malt1*-PD and -PDT mice.

CTLA-4 expression on T cells depends on mTORC1 activation downstream of TCR stimulation (74). Of interest, mTORC1 activation was recently shown to require MALT1 protease activity via regulation of the glutamine import protein ASCT2 (75, 76). Reduced CTLA-4 expression on Tregs and effector CD4^+^ T cells from *Malt1*-PDT mice might therefore reflect reduced mTORC1 activation. Moreover, since CTLA-4 mRNA is directly targeted for degradation by the MALT1 substrates Regnase-1, Roquin-1, and Roquin-2 (37), reduced CTLA-4 expression on Tregs and effector CD4^+^ T cells from *Malt1*-PDT mice may also reflect its post-transcriptional regulation by MALT1. Based on our results, we propose that future risk assessments of MALT1 protease inhibition related to autoimmunity could focus on the effect of MALT1 inhibitors on Treg differentiation, maintenance and functionality. It should be mentioned that there is an important difference between genetic and pharmacological approaches to assess the role of MALT1 proteolytic activity in immune homeostasis. In case of genetic inactivation, MALT1 function is disrupted already in the germline (in case of full-body KI mice) or is removed during early T cell differentiation (in case of conditional *CD4-Cre* KI mice). In contrast, upon pharmacological inhibition with for example small compound inhibitors of MALT1, Tregs are MALT1 protease proficient before the treatment is started, which may result in a different outcome. While the current paper was written, however, several studies using *Malt1*^FL/PD^ *Foxp3-Cre*^*Tg*/+^ mice that specifically express catalytically inactive MALT1 in Tregs indicated that post-development loss of MALT1 proteolytic activity in Tregs leads to severe autoimmune disease (77–79), which might raise concerns regarding the safety of pharmacological inhibition of MALT1 protease activity. Based on our results on CTLA-4 and the above mentioned other studies, it would be highly interesting to see whether *Foxp3-Cre* conditional KO of *Regnase-1* and/or *Roquin-1* and *Roquin-2* can rescue the autoimmune disease in *Malt1*-PD mice. Noteworthy, a MALT1 protease inhibitor will dampen both effector T cells and Tregs, which can still lead to a different immune homeostasis phenotype compared to the autoimmune phenotype upon Treg-specific loss of MALT1 proteolytic activity. Importantly, there is already indirect evidence indicating that therapeutic targeting of MALT1 protease activity in adult mice with small compound inhibitors is safe. Firstly, extended treatment of mice with the MALT1 protease inhibitor mepazine did not reduce Treg numbers (40). As a counterexample, long-term treatment of psychiatric patients with chlorpromazine, another MALT1 inhibitor, is associated with the induction of autoimmunity markers and occasionally results in a lupus-like disease (80, 81). It is however important to stress that both these phenothiazine compounds also have other MALT1-independent activities that can be responsible for the observed effects (82). Another argument in favor of safety for MALT1 inhibitor treatment in adults is the fact that MALT1 seems to be specifically required for the development of nTregs, which develop at a young age in the thymus (54). A third compelling argument is that only a small amount of functional (WT) Tregs is enough to suppress the autoimmune phenotype of *Malt1*-PD mice (5). Since therapeutic inhibition of enzymatic activity is never complete and can be adjusted by dosage and treatment regimes, it might be that the remaining functional Tregs are enough to prevent autoimmunity. Nevertheless, a major future challenge will be to generate and characterize inducible genetic models to emulate the effects of long-term inhibitor treatment in adults.

## Materials and Methods

### Mouse *Rosa26*^*LSL-hMALT1-WT*^ Transgenesis

#### Cloning of RMCE-Targeting Constructs

Plasmids of the cloned genes were deposited in the BCCM/GeneCorner plasmid collection along with detailed descriptions of cloning strategy and plasmid sequence (http://www.genecorner.ugent.be/). Human *MALT1* (LMBP 9104, LMBP 9105) was cloned into pENTR221 together with a genotype-specific barcode sequence downstream of the stop codon for genotyping. The ENTR clones were integrated in the pRMCE_DV1 (LMBP 8870) destination vector to generate RMCE targeting vectors for *MALT1* (LMBP 9111, LMBP 9112).

#### RMCE Targeting

RMCE compatible mouse ES-cells [G4 ROSALUC (83)] were cultured in standard ES-cell medium containing 500 ml KO-DMEM (Thermo Fisher Scientific), 15% serum (Hyclone), 100 μM Non-essential amino acids (Thermo Fisher Scientific), 1x Glutamax (Thermo Fisher Scientific), 100 μM β-mercaptothanol (Thermo Fisher Scientific) and 2,000 units LIF/ml (VIB Protein Core). ES-cells were co-transfected with 2.5 μg of a FlpE expressing plasmid (84) and 2.5 μg of targeting vector using lipofectamin 2000 (Thermo Fisher Scientific) according to the manufacturer's instructions. G418 selection (200 μg/ml) was applied 48 h after transfection. After 7–10 days, individual G418 resistant, RMCE targeted ES cell clones were picked and further expanded. Correct targeting events were confirmed by Southern blot analysis (5′ probe, EcoRI, wt = 15,5 kb/mutant = 4.5 kb/wtRosaluc allele = 6 kb; EGFP internal probe, KpnI, single integration = 11 kb) and by PCR; the targeted allele generates a band of 560 bp (primers: FW 5′ AAA GTC GCT CTG AGT TGT TAT 3′ and REV 5′ GCG GCC TCG ACT CTA CGA TA 3′). Correctly targeted ES-cells were aggregated with outbred Swiss morula, which were then implanted into pseudopregnant Swiss mice.

### Mice

In order to generate conditional *Malt1*-KO mice, mice were generated from the EUCOMM *Malt1*^tm1a(EUCOMM)Hmgu/+^ ES cells (85), which were back-crossed to a germ line Flp deleter (86) to generate *Malt1*^*FL*/+^ mice. These mice were further crossed to the *CD4-Cre* (87) line and back-crossed to generate Flp-negative T cell-specific *Malt1*-KO (*Malt1*^*FL*/*FL*^ *CD4-Cre*) (88–91). The *Malt1*^*tm1a*(*EUCOMM*)*Hmgu*/+^ mice were also back-crossed with a germline Cre-line and back crossed to generate Cre-negative co-isogenic *Malt1*^−*LacZ*/−*LacZ*^ full-body KO mice. *Malt1*^*PD*/+^ (the mouse MALT1 C472A mutation that disrupts protease activity) mice were generated in a C57/BL6 background by CRISPR/Cas9 (92) by Cyagen Biosciences Inc. *Malt1-*PD mice were always maintained as heterozygotes (*Malt1*^*PD*/+^) in breeding to avoid suffering from the autoimmune phenotype. *Rosa26*^*LSL-MALT1-WT*^ transgenic mice were crossed with CD4-Cre and subsequently back-crossed with full-body *Malt1* KO mice, and later back-crossed to *Malt1*^*PD*/+^ mice to generate rescued *Malt1*^*PD*/−^ *Rosa26*^*LSL*−*MALT*1−*WT*^ *CD4-Cre* and *Malt1*^*PD*/*PD*^ *Rosa26*^*LSL*−*MALT*1−*WT*^ *CD4-Cre* mice where all cells except T cells are MALT1 protease deficient. The transgenes were confirmed by western blot (Supplemental Figure 4) for specific expression of MALT1 only in presence of CD4-Cre using a human-specific rabbit monoclonal anti-MALT1 antibody (EP603Y, Abcam), anti-mouse MALT1 (sc-28246, Santa Cruz) and anti-Cre (69050-3, Merck Millipore). Mice used in these experiments were kept at room temperature, fed *ad libitum* in an SPF facility (IRC, VIB-UGent). All experiments were done according to the UGent ethical guidelines with the local ethical committee approval number: EC 2015-031.

### Genotyping

The *Malt1* Flox-allele or KO allele from Eucomm was monitored with the primers MALTcKO-F (GTTTCTCAGGTCTTTAGTTCATGTC), CoMLT-3-R (TATACTCTACATCTCCATGGT), MALTcKO-R (TTGTTTTGCAGATCTCTGCC), and MLT-LacZ-F (TCGCTACCATTACCAGTTGGT) resulting in 280 bp (WT), 400 bp (FL), 345 (KO), or 514 bp (KO-LacZ) PCR products. SNP-specific primers were designed as described in Staal et al. (93), with the allele-specific nucleotides close to the 3′ end of the primer. The *Malt1* C472A KI was monitored with the primers F-MALT-KICA-GT (CCCACTCCCAGGATTGTTATATT), R-MALT1-KICA-GT (TGCTCTAGATCCACAGGTGTGGTT), KI-MALT-CA-F (AATGTGTTCCTGTTGGATATGGCCAG), and KI-MALT-WT-R (GAGACATTTTACCTTTTCCGACAC) resulting in 461 bp (allele-independent), 304 bp (PD) and 201 bp (WT) PCR fragments. The *Rosa26*^*LSL-MALT1-WT*^ transgenes were genotyped with the primers RMCE-MALT-F (GCAGCTACTTGGTATCAAAGGATCT), RMCE-MALT-R (TTTCCACAACTATCCATGTCGC), generating an allele-independent 896 bp PCR fragment, and the allele-specific primer RMCE-MALT-WT-R (CCAATTTCTGAAAAATAGTGTCCAAC) generating an allele specific band of 537 bp. Flp was detected with the primers Flp-F (TTAGTTCAGCAGCACATGATG) and Flp-R (GGAGGATTTGATATTCACCTG), resulting in a 370 bp PCR fragment. Cre was detected with the primers Cre-F (TGCCACGACCAAGTGACAGCAATG) and Cre-R (AGAGACGGAAATCCATCGCTCG) producing a 374 bp PCR fragment. All genotypings were done using the GoTaq Green Hot Start (Promega) master mix, with a typical PCR program: 5 min 95°C denaturation, 35–40 cycles [30 s 95°C|30 s 52–60°C|60 s 72°C] and 10 min 72°C final elongation.

### Flow Cytometry

Data were obtained with a LSRII flow cytometer (BD Biosciences) and FlowJo Software (Treestar, Inc., Ashland, Ore) was used for data analysis.

#### Detection of Tregs

Cells from thymus and cLN were surfaced stained with Fixable Viability Dye eFluor506 (eBioscience), anti-CD16/CD32 Fc block (clone 2.4G2; BD Biosciences), anti-CD3-eFluor450 (clone 17A2; eBioscience), anti-CD4-FITC (clone GK1.5; BD Biosciences or eBioscience), anti-CD25-PercPcy5.5 (clone PC61; BD Biosciences) for 20 min. Next, cells were permeabilized for 30 min, followed by 30 min of intracellular staining for anti-Foxp3-PE (clone FJK-16s; eBioscience). For the intracellular staining, the Foxp3 buffer set (eBioscience) was used and all incubation steps were done on ice.

#### Analysis of Effector Markers on Tregs

Cells from cLN cultured in complete medium (RPMI 1640 medium supplemented with 10% FCS, sodium pyruvate, L-glutamine, antibiotics and β-mercaptoethanol) were stimulated with PMA (50 ng/ml) and ionomycin (1 μg/ml) for 4 h at 37°C. The cells were stained as mentioned above, but anti-CD44-APC-efluor780 (clone IM7; eBioscience) and anti-CTLA-4 PE-eFluor610 (clone UC10-4B9; eBioscience) were also included in the surface staining. For the other Treg effector markers, non-stimulated cells from cLN were stained as mentioned above, but anti-CD44-AlexaFluor700 (clone IM7; BD Pharmingen), anti-CD62L-eFluor450 (clone MEL-14; eBioscience) and anti-KLRG1-APC (clone 2F1; eBioscience) or anti-TNFR2-BV421 (clone TR75-89; BD Pharmingen) and anti-ICOS-PE-Cy5 (clone 7E.17G9; eBioscience) were also included in the surface stain.

#### Analysis of Cytokines by Intracellular Cytokine Staining

Cells from cLN were cultured in complete medium and stimulated with PMA (50 ng/ml), ionomycin (500 ng/ml) and brefeldin A (1 μg/ml) for 4–5 h at 37°C. Stimulated cells were washed, surface stained with anti-CD16/CD32, Fixable Viability Dye eFluor 506, anti-CD3-eFluor450, anti-CD4-FITC, anti-CD44-APCeFluor780, for 20 min. Next, cells were fixed and permeabilized for 30 min using the Foxp3 buffer set, followed by intracellular staining with anti-IFNγ-PE-Cy7 (clone XMG1.2; BD Pharmingen) for 30 min.

#### Detection of MZ B Cells

Spleen cells were surface stained with Fixable Viability Dye eFluor506 (eBioscience), anti-CD16/CD32 Fc block (clone 2.4G2; BD Biosciences), anti-CD3-PE-Cy5 (145-2C11; Tonbo Biosciences), anti-CD45R/B220 (clone RA3-6B2; BD Biosciences), anti-CD21/CD35-eFluor450 (clone 4E3; eBioscience), and anti-CD23-PE-Cy7 (clone B3B4,eBioscience) for 20 min.

### Cytokine Analysis

Peripheral blood samples were collected for serum preparation. The levels of IL-2 (171-G5003M), IL-4 (171-G5005M), IL-6 (171-G5007M), IL-17 (171-G5013M), IFN-γ (171-G5017M), and TNF (171-G5023M) were determined by Bio-Plex (Biorad) according to the manufacturer's conditions. B cells were purified from spleen with the MagniSort Mouse B cell Enrichment Kit (eBioscience), cultured in complete medium (RPMI 1640 medium supplemented with 10% FCS, sodium pyruvate, L-glutamine, antibiotics and β-mercaptoethanol) and stimulated overnight with 20 μM CpG1826 (Invivogen) as described (47). The level of IL-10 (171G5009M) in medium was determined by Bio-Plex (Biorad) according to manufacturer's conditions.

### Histology

Stomach, lacrimal gland and salivary gland were fixed with 4% paraformaldehyde and imbedded in paraffin. Sections (5 μm) were stained with haematoxylin and eosin. Images (100× magnification) were acquired with a BX51 discussion microscope (Olympus) with PixeLink camera under 100× magnification.

## Data Availability

All datasets generated for this study are included in the manuscript/Supplementary Files.

## Ethics Statement

All experiments were done according to the UGent ethical guidelines with the local ethical committee approval number: EC 2015-031.

## Author Contributions

AD and JS designed the experiments. AD performed all experiments, except for the weighing and ataxia scoring done by JS, YD, and AD. IS did experiments for MZ B cells. YD and MK assisted with genotyping of mice. The *Malt1*^*PD*/+^ C472A PD mice were generated by MB. TH generated the *Rosa26*^*LSL*−*hMALT*1−*WT*^ transgenic mice and the *Malt1*^*tm*1*a*(*EUCOMM*)*Hmgu*/+^ mice from EUCOMM ES cells. AD, JS, MB, and RB contributed to the scientific discussion. AD, JS, and RB wrote the manuscript.

### Conflict of Interest Statement

The authors declare that the research was conducted in the absence of any commercial or financial relationships that could be construed as a potential conflict of interest. Part of the work was done during a research collaboration between VIB, CD3, and AstraZeneca on MALT1 inhibitors (94).
