# Supplementary material for: MALT1 Proteolytic Activity Suppresses Autoimmunity in a T Cell Intrinsic Manner
Source: Front Immunol. 2019 Aug 14;10:1898. doi: 10.3389/fimmu.2019.01898 (PMC6702287; doi:10.3389/fimmu.2019.01898)
Supplement: Supplementary file 1 [file Image_1.pdf]

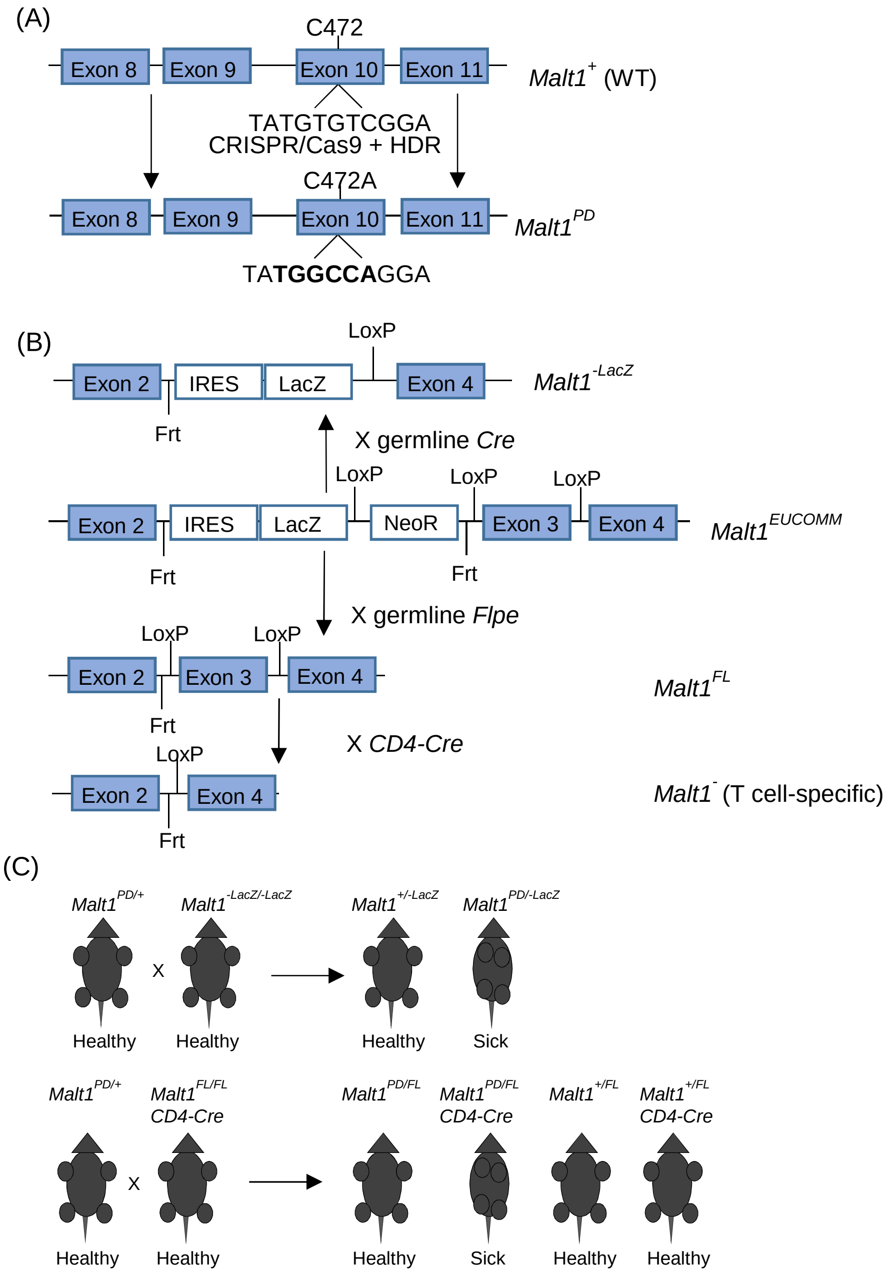

Supplementary Figure 1: Overview of the strategy used to generate different mouse lines

(**A**) Generation of the *MALT1*-PD mice carrying a C472A mutation in exon 10 (ENSMUSE00000318133) by CRISPR/Cas9 and homology directed repair. The mutation introduces a silent MscI (TGG|CCA) restriction site. **(B)** Generation of *Malt1*$^{-Lacz}$ mice from the original EUCOMM insert. The neomycin resistance cassette and exon 3 are removed by germline *Cre* recombinase expression, but the *E. coli* galactosidase (LacZ) reporter is maintained. Generation of conditional *Malt1*$^{FL/FL}$ KO mice from the original EUCOMM insert. The LacZ reporter and the neomycin resistance cassette is removed by germline *Flp* recombinase expression, leaving exon 3 (ENSMUSE00000318222) flanked by loxP sites. Upon Cre recombinase expression (either germ-line or cell-specific), the 122 bp exon 3 is deleted causing a -1 frame shift and KO of *Malt1*. (**C**) Healthy *Malt1*$^{PD/+}$ mice are crossed with either full *Malt1*-KO (*Malt1*$^{-LacZ/-LacZ}$) mice, which causes a 1:1 ratio of healthy and sick offspring, or with T cell-specific *Malt1*-KO (*Malt1*$^{FL/FL}$ *CD4-Cre*) mice, which causes a 3:1 ratio of healthy vs sick offspring.

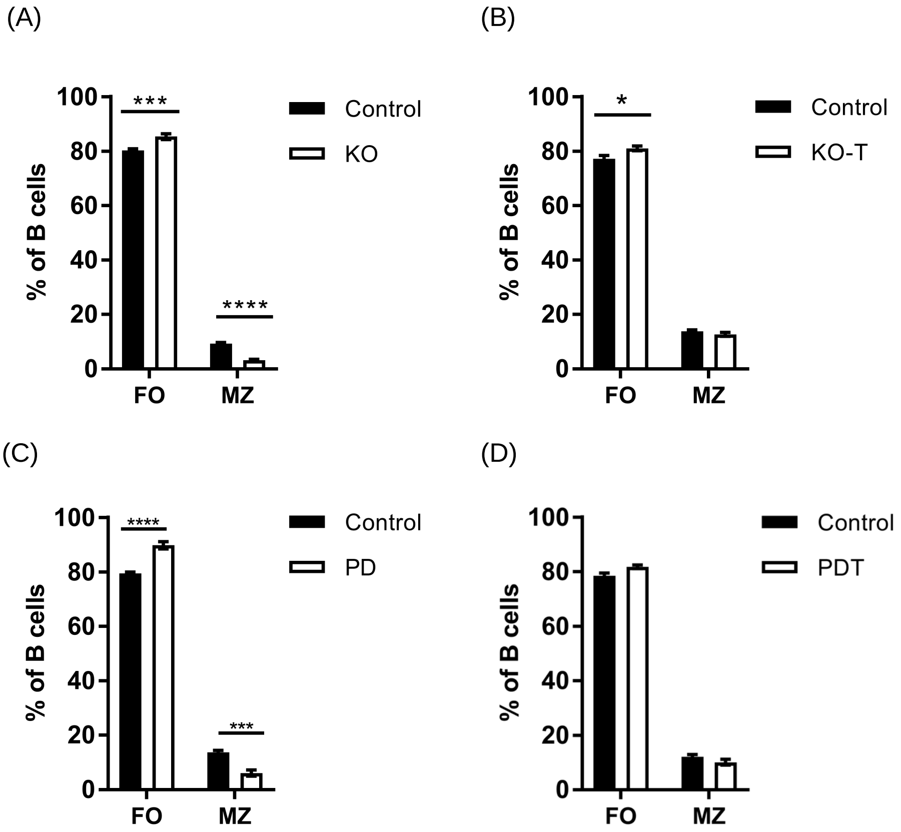

Supplementary Figure 2: MZ B cells are reduced in *Malt1*-KO and *Malt1*-PD mice but not in T cell-specific *Malt1*-KO and *Malt1*-PDT mice

(A-D) Frequency of MZ B cells (% CD21$^+$CD23$^-$ of B220$^+$ B cells) and follicular (FO) B cells (% CD21$^+$CD23$^+$ of B220$^+$ B cells) in the spleen of *Malt1*-KO **(A)**, *Malt1$^{FL/FL}$ CD4-Cre* (KO-T) **(B)**, *Malt1*-PD **(C)** and *Malt1*-PDT mice **(D)** that suffer from ataxia, and corresponding control mice. Data information: All data were obtained via flow cytometry. **(A)** *Malt1*-KO mice n = 5 and corresponding control mice n = 4. **(B)** *Malt1$^{FL/FL}$ CD4-Cre* (KO-T) mice n = 5 and corresponding control mice n = 3. **(C)** *Malt1*-PD mice n = 4 and corresponding control mice n = 6. **(D)** *Malt1*-PDT mice n =3 and corresponding control mice n = 6. The mean +/- SEM is indicated on the graphs. The statistical significance between groups was calculated with an unpaired 2 tailed Student's *t*-test: * $P < 0,05$, **$P < 0.01$, *** $p < 0,001$ and **** $p < 0.0001$.

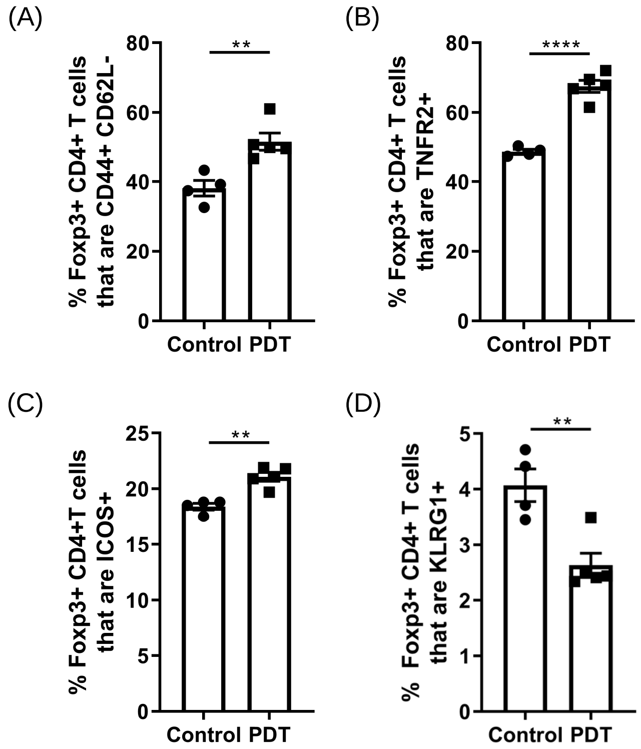

Supplementary Figure 3: *Malt1*-PDT mice do not have a generalized defect in Treg effector markers

(A) Frequency of effector Tregs (% $CD44^{+}CD62L^{-}$ of $Foxp3^{+}CD4^{+}$ T cells) and **(B-D)** frequency of Tregs expressing TNFR2, ICOS or KLRG1 on their surface in cLN of 3-4 week old *Malt1*-PDT (n = 5) and corresponding control mice (n = 4). All data were obtained via flow cytometry. The mean +/- SEM is indicated on the graphs. The statistical significance between groups was calculated with an unpaired 2 tailed Student's t-test: ** $p < 0.01$ and **** $p < 0.0001$.

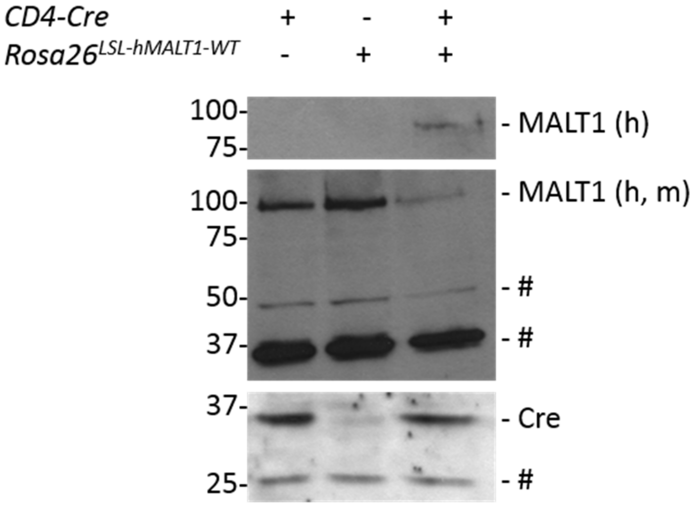

Supplementary Figure 4: Western blot showing *CD4-Cre*-inducible hMALT1 transgene expression

Splenocytes from *Malt1*$^{PD/-}$ *CD4-Cre*, *Malt1*$^{PD/-}$ *Rosa26*$^{LSL\text{-}hMALT1\text{-}WT}$ and *Malt1*$^{PD/-}$ *CD4-Cre Rosa26*$^{LSL\text{-}hMALT1\text{-}WT}$ mice were lysed in E1A buffer and equal amounts of protein (100µg) were run on SDS-PAGE and blotted. The western blot was developed with a human-specific anti-MALT1 antibody (top panel), a human/mouse specific anti-MALT1 antibody (middle panel), and an anti-Cre recombinase antibody (lower panel). Aspecific bands (#) are used as loading control.
